# Na_3_Co_2_(AsO_4_)(As_2_O_7_): a new sodium cobalt arsenate

**DOI:** 10.1107/S1600536812027791

**Published:** 2012-06-27

**Authors:** Abderrahmen Guesmi, Ahmed Driss

**Affiliations:** a Laboratoire de Matériaux et Cristallochimie, Faculté des Sciences, El Manar II, 2092 Tunis, Tunisia; b Institut Préparatoire aux Etudes d’Ingénieurs d’El Manar, BP 244 El Manar II, 2092 Tunis, Tunisia

## Abstract

In the title compound, tris­odium dicobalt arsenate diarsenate, Na_3_Co_2_AsO_4_As_2_O_7_, the two Co atoms, one of the two As and three of the seven O atoms lie on special positions, with site symmetries 2 and *m* for the Co, *m* for the As, and 2 and twice *m* for the O atoms. The two Na atoms are disordered over two general and special positions [occupancies 0.72 (3):0.28 (3) and 0.940 (6):0.060 (6), respectively]. The main structural feature is the association of the CoO_6_ octa­hedra in the *ab* plane, forming Co_4_O_20_ units, which are corner- and edge-connected *via* AsO_4_ and As_2_O_7_ arsenate groups, giving rise to a complex polyhedral connectivity with small tunnels, such as those running along the *b*- and *c*-axis directions, in which the Na^+^ ions reside. The structural model is validated by both bond-valence-sum and charge-distribution methods, and the distortion of the coordination polyhedra is analyzed by means of the effective coordination number.

## Related literature

For related structures, see: Ruiz-Valero *et al.* (1996 ▶); Ben Smail & Jouini (2005 ▶); Guesmi & Driss (2002*a* ▶,*b* ▶). For bond-valence analysis, see: Brown (2002 ▶); Adams (2003 ▶). For the charge distribution method, see: Nespolo *et al.* (2001 ▶); Nespolo (2001 ▶); Guesmi *et al.* (2006 ▶).

## Experimental

### 

#### Crystal data

Na_3_Co_2_(AsO_4_)(As_2_O_7_)
*M* *_r_* = 587.59
Monoclinic, 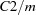

*a* = 10.484 (3) Å
*b* = 16.309 (2) Å
*c* = 6.531 (1) Å
β = 120.40 (2)°
*V* = 963.2 (3) Å^3^
*Z* = 4
Mo *K*α radiation
μ = 13.87 mm^−1^
*T* = 293 K
0.20 × 0.10 × 0.10 mm

#### Data collection

Enraf–Nonius CAD-4 diffractometer
Absorption correction: ψ scan (North *et al.*, 1968 ▶) *T*_min_ = 0.168, *T*_max_ = 0.338
1765 measured reflections
1183 independent reflections
998 reflections with *I* > 2σ(*I*)
*R*_int_ = 0.041
2 standard reflections every 120 min intensity decay: 1%

#### Refinement

*R*[*F* ^2^ > 2σ(*F* ^2^)] = 0.027
*wR*(*F* ^2^) = 0.067
*S* = 1.01
1183 reflections
103 parameters
Δρ_max_ = 0.72 e Å^−3^
Δρ_min_ = −0.80 e Å^−3^

### 

Data collection: *CAD-4 EXPRESS* (Enraf–Nonius, 1995 ▶); cell refinement: *CAD-4 EXPRESS*; data reduction: *XCAD4* (Harms & Wocadlo, 1995 ▶); program(s) used to solve structure: *SHELXS97* (Sheldrick, 2008 ▶); program(s) used to refine structure: *SHELXL97* (Sheldrick, 2008 ▶); molecular graphics: *DIAMOND* (Brandenburg, 2001 ▶); software used to prepare material for publication: *WinGX* (Farrugia, 1999 ▶) and *publCIF* (Westrip, 2010) ▶.

## Supplementary Material

Crystal structure: contains datablock(s) I, global. DOI: 10.1107/S1600536812027791/jj2142sup1.cif

Structure factors: contains datablock(s) I. DOI: 10.1107/S1600536812027791/jj2142Isup2.hkl

Supplementary material file. DOI: 10.1107/S1600536812027791/jj2142Isup3.cml

Additional supplementary materials:  crystallographic information; 3D view; checkCIF report

## Figures and Tables

**Table 1:** Bond-valence-sum and charge distribution analysis

Cation	*q*(*i*)·*sof*(*i*)	*V*(*i*)	*Q*(*i*)	CN(*i*)	ECoN(*i*)	*d*_moy_(*i*)	*d*_med_(*i*)
Co1	2.00	1.87	2.01	6	5.97	2.14	2.14
Co2	2.00	2.05	2.03	6	5.62	2.11	2.08
As1	5.00	4.99	5.13	4	3.99	1.69	1.69
As2	5.00	4.93	4.94	4	3.89	1.70	1.69
Na1*A*	0.72	0.65	0.72	8	7.33	2.67	2.63
Na1*B*	0.28	0.25	0.27	8	5.72	2.74	2.58
Na2*A*	0.94	1.02	0.92	7	5.52	2.58	2.45
Na2*B*	0.06	0.07	0.06	6	4.78	2.45	2.31

*q*(*i*) = formal oxidation number; *sof*(*i*) = site occupation factor; *d*_moy_(*i*) = arithmetic average distance (Å); *d*_med_(*i*) = weighted average distance (Å); sodium CNS for *d*(Na—O)_max_ = 3.10 Å; σ_cat_ = dispersion factor on cationic charges measuring the deviation of the computed charges (*Q*) with respect to the formal oxidation numbers; σ_cat_ = [Σ_*i*_(*q*_*i*_−*Q*_*i*_)^2^/*N*−1]^1/2^ = 0.055.

**Fig. 1.**
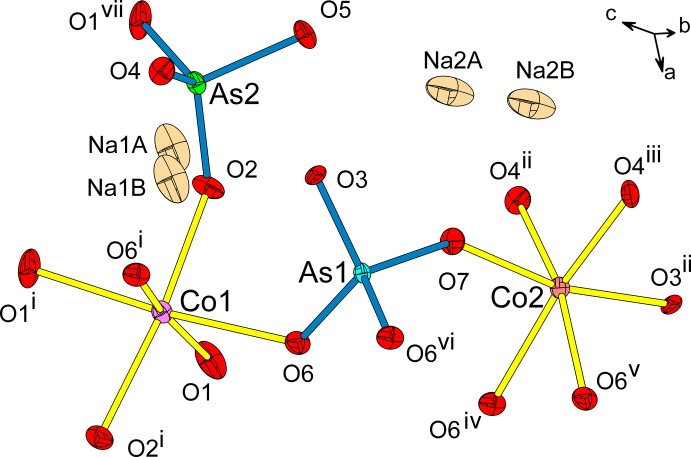
The asymmetric unit in (I) with atom-labelling scheme. Some symmetry-related O atoms are included to show the full coordination polyhedra around the Co and As atoms. Displacement ellipsoids are drawn at the 50% probability level [Symmetry codes: (i) -x+1, y, -z+2; (ii) x, y, z-1; (iii) x, -y+1, z-1; (iv) -x+1, y, -z+1; (v) -x+1, -y+1, -z+1; (vi) x, -y+1, z; (vii) x-1/2, -y+1/2, z.]

**Fig. 2.**
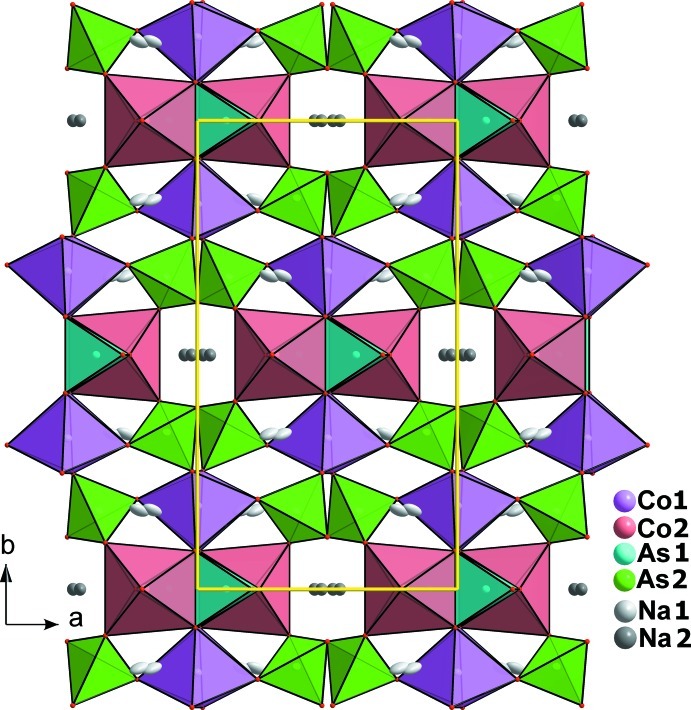
Polyhedron framework structure of (I) viewed along the c axis.

**Fig. 3.**
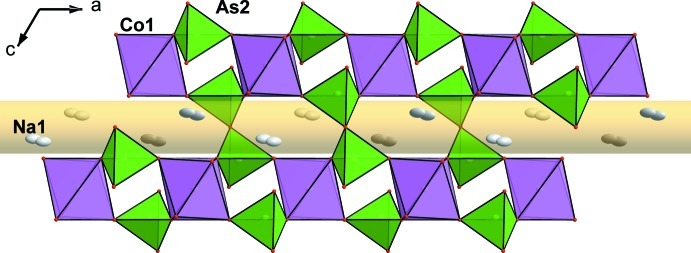
The polyhedral layers in the framework of (I); Na1 cations are on the periphery of tunnels parallel to [100].

**Fig. 4.**
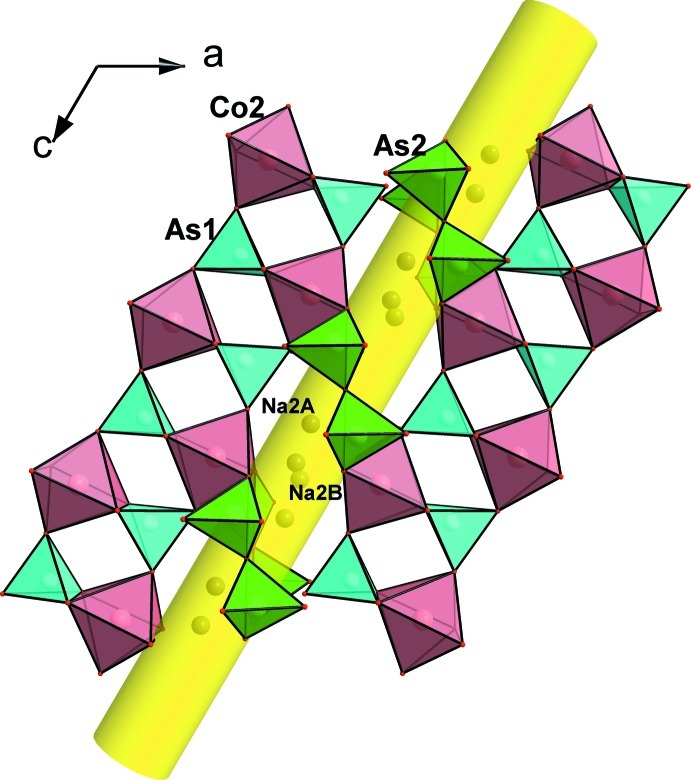
The connection between the chains parallel to [001]; Na2 cations are inside the resulted tunnels.

## supplementary crystallographic information

### Comment

The rich chemistry of the A–Co–P/As–O crystallographic systems (A is a monovalent cation), has been shown by the synthesis and crystal structures of several compounds with particular crystallographic properties such as: Na_4_Co_3_(PO_4_)_2_P_2_O_7_, a phosphate containing a three-dimensional system of large intersecting tunnels (Ruiz-Valero *et al.* 1996), AgCo_3_H_2_(PO_4_)_3_, an alluaudite-like phosphate structure (Guesmi & Driss, 2002*a*), K_2_CoP_2_O_7_, a layered tetrahedral phosphate with the mellilite structure (Guesmi & Driss, 2002*b*), *etc*.

For the case of arsenates, their main structural difference if compared to phosphates is that arsenic atoms can also adopt an octahedral coordination; it is the case for example of the oxygen-deficient layered sodium arsenate Na_7_As_11_O_31_ (Guesmi *et al.* 2006). Continuous investigations on the crystal chemistry of the arsenates are performed because arsenic is at the top of the priority of the most hazardous substances, but less is known about its crystal structures.

We are interested in the present work in the crystal structure of the new compound Na_3_Co_2_AsO_4_As_2_O_7_ (I). The crystal structure of the isostructural Na_3_Ni_2_(As_0.1_P_0.9_)O_4_(As_1.3_P_0.7_)O_7_ compound and ionic conductivity properties of its limiting arsenate has been studied (Ben Smail & Jouini, 2005). The chemical formula of (I) has been established as a result of the crystal structure determination and the obtained structural model is validated by means of charge distribution (CD) (Nespolo *et al.* 2001, Nespolo, 2001) and bond valence sum methods (BVS) (Brown, 2002; Adams, 2003) as the formal charges (Q) and valences (V) agree well with the expected values (Table 1).

The new compound (I) is an example of a mixed transition-metal arsenate, representing the first cobalt arsenate built up from mono- and diarsenate groups. In the asymetric unit, the crystal structure is built up from corner and edge-sharing between cobalt octahedra and arsenate groups (Fig. 1). The two crystallographically distinct cobalt atoms exhibit a slightly distorted octahedral coordination with effective coordination numbers ECoN(Co1)=5.97 and ECoN(Co2)=5.62 and weighted average distances d_med_(Co1)=2.14 Å and d_med_(Co2)=2.08 Å. The longest Co–O6 bond distances in the two octahedra correspond to the three-coordinated oxygen atom, related also to As1.

The As1 tetrahedron, with a 2 + 2 coordination, shares its four corners with five octahedra. The As2 tetrahedron, a more precisely a trigonal pyramid (1 + 3 coordination), is more distorted with O5 as a bridging oxygen in the As(2)_2_O_7_ group (ECoN(As2)=3.89 and d_med_(As2)=1.69 Å). The other six corners in the diarsenate group are common with four Co1 and two Co2 octahedra. It is worth noting that the Co2 and As1 polyhedra share a common edge which induces a strong repulsion between positive charges; this type of connection was also observed in the structure of Na_4_Co_3_(PO_4_)_2_P_2_O_7_ (Ruiz-Valero *et al.* 1996).

The cobalt octahedra are associated in the *ab* plane to form the original octahedral metallic units Co_4_O_20_ which are corner- and edge-connected *via* As(1)O_4_ and As(2)_2_O_7_ arsenate groups, giving rise to a complex polyhedral connectivity which produces small tunnels, such as those running along the *b* and *c* axis, where the sodium cations reside (Figs. 2–4).

The anionic framework can be decomposed in a succession of alternate layers in the *ac* plane, stacked along the crystallographic *b*-axis. They are built up of Co1 octahedra and As(2)_2_O_7_ groups in such a way that each octahedron is corner-shared to four diarsenate groups (Fig. 3). These layers are alternate by a chain type resulting from the connection between Co2 and As1 polyhedra and formed by the centrosymmetric cyclic units [Co_2_As_2_O_14_] (Fig. 4), each one of these units is connected to two neighbours by means of mixed Co–O–As bridges.

The Na1 ions are split into two independent positions near *c/2*, Na1B has the more distorted polyhedron and the ECoN(Na1B) is as low as 5.72. The Na2 ions are also disordered with the Na2B polyhedron sandwiched by Na2A ones which are off-centred around the Na2B positions. The motion of sodium cations within the framewok of (I) by means of theoretical studies and electrical measuremeents will be the subject of future works.

### Experimental

The investigated compound was synthesized by a solid state reaction from a mixture of Na_2_CO_3_ (0.46 g, Fluka, 99.0%), cobalt (II and III) oxides (0.1 g, Fluka, 99.0%, Co 71% min.) and As_2_O_5_ (0.33 g, Prolabo). The reaction mixture was heated at 673 K for 24 h and progressively at 923 K and kept at this temperature for three days. Finally, it was slowly cooled to room temperature. The obtained pink crystals were separated from the excess flux by washing the product in boiling water.

### Refinement

The non-equivalent sodium ions are inserted in the anionic framework first in two full-occupied general and special crystallographic sites. The Na1 atoms are better described by a split model with two independent general positions, refined with the same thermal paramaters. The highest Fourier peaks near the Na2A site suggests that the Na2A position deviates from the full occupancy and another partial-occupied position (Na2B) was introduced in the model, leading to a lowering of *R* values and residual electron density peaks.

### Crystal data

**Table:**

Na_3_Co_2_(AsO_4_)(As_2_O_7_)	*F*(000) = 1096
*M**_r_* = 587.59	*D*_x_ = 4.052 Mg m^−^^3^
Monoclinic, *C*2/*m*	Mo *K*α radiation, λ = 0.71073 Å
Hall symbol: -C 2y	Cell parameters from 25 reflections
*a* = 10.484 (3) Å	θ = 11.7–14.5°
*b* = 16.309 (2) Å	µ = 13.87 mm^−^^1^
*c* = 6.531 (1) Å	*T* = 293 K
β = 120.40 (2)°	Parallelepiped, pink
*V* = 963.2 (3) Å^3^	0.20 × 0.10 × 0.10 mm
*Z* = 4	

### Data collection

**Table:**

Enraf–Nonius CAD-4 diffractometer	998 reflections with *I* > 2σ(*I*)
Radiation source: fine-focus sealed tube	*R*_int_ = 0.041
Graphite monochromator	θ_max_ = 28.0°, θ_min_ = 2.5°
ω/2θ scans	*h* = −13→13
Absorption correction: ψ scan (North *et al.*, 1968)	*k* = −1→21
*T*_min_ = 0.168, *T*_max_ = 0.338	*l* = −8→3
1765 measured reflections	2 standard reflections every 120 min
1183 independent reflections	intensity decay: 1%

### Refinement

**Table:**

Refinement on *F*^2^	Primary atom site location: structure-invariant direct methods
Least-squares matrix: full	Secondary atom site location: difference Fourier map
*R*[*F*^2^ > 2σ(*F*^2^)] = 0.027	*w* = 1/[σ^2^(*F*_o_^2^) + (0.0261*P*)^2^] where *P* = (*F*_o_^2^ + 2*F*_c_^2^)/3
*wR*(*F*^2^) = 0.067	(Δ/σ)_max_ = 0.005
*S* = 1.01	Δρ_max_ = 0.72 e Å^−^^3^
1183 reflections	Δρ_min_ = −0.80 e Å^−^^3^
103 parameters	Extinction correction: *SHELXL97* (Sheldrick, 2008), Fc^*^=kFc[1+0.001xFc^2^λ^3^/sin(2θ)]^-1/4^
0 restraints	Extinction coefficient: 0.00124 (17)

### Special details

**Table:**

Geometry. All e.s.d.'s (except the e.s.d. in the dihedral angle between two l.s. planes) are estimated using the full covariance matrix. The cell e.s.d.'s are taken into account individually in the estimation of e.s.d.'s in distances, angles and torsion angles; correlations between e.s.d.'s in cell parameters are only used when they are defined by crystal symmetry. An approximate (isotropic) treatment of cell e.s.d.'s is used for estimating e.s.d.'s involving l.s. planes.
Refinement. Refinement of *F*^2^ against ALL reflections. The weighted *R*-factor *wR* and goodness of fit *S* are based on *F*^2^, conventional *R*-factors *R* are based on *F*, with *F* set to zero for negative *F*^2^. The threshold expression of *F*^2^ > σ(*F*^2^) is used only for calculating *R*-factors(gt) *etc*. and is not relevant to the choice of reflections for refinement. *R*-factors based on *F*^2^ are statistically about twice as large as those based on *F*, and *R*- factors based on ALL data will be even larger.

### Fractional atomic coordinates and isotropic or equivalent isotropic displacement parameters (Å^2^)

**Table:**

	*x*	*y*	*z*	*U*_iso_*/*U*_eq_	Occ. (<1)
Co1	0.5000	0.32196 (5)	1.0000	0.0091 (2)	
Co2	0.29864 (9)	0.5000	0.08408 (15)	0.0090 (2)	
As1	0.38970 (6)	0.5000	0.67693 (11)	0.00692 (16)	
As2	0.11973 (5)	0.33430 (3)	0.76867 (8)	0.00892 (14)	
O1	0.5206 (4)	0.2480 (2)	0.7535 (6)	0.0219 (8)	
O2	0.2686 (3)	0.3079 (2)	0.7525 (6)	0.0173 (7)	
O3	0.2608 (5)	0.5000	0.7550 (8)	0.0097 (9)	
O4	0.1460 (3)	0.40124 (19)	0.9821 (6)	0.0136 (6)	
O5	0.0000	0.3884 (3)	0.5000	0.0133 (9)	
O6	0.5077 (3)	0.41858 (19)	0.7766 (6)	0.0126 (6)	
O7	0.2869 (5)	0.5000	0.3808 (8)	0.0193 (11)	
Na1A	0.1740 (18)	0.1692 (4)	0.5940 (15)	0.032 (2)	0.72 (3)
Na1B	0.225 (3)	0.1686 (13)	0.617 (4)	0.032 (2)	0.28 (3)
Na2A	0.0488 (3)	0.5000	0.2908 (8)	0.0353 (11)	0.940 (6)
Na2B	−0.021 (6)	0.5000	−0.054 (13)	0.0353 (11)	0.060 (6)

### Atomic displacement parameters (Å^2^)

**Table:**

	*U*^11^	*U*^22^	*U*^33^	*U*^12^	*U*^13^	*U*^23^
Co1	0.0097 (4)	0.0075 (4)	0.0088 (4)	0.000	0.0037 (3)	0.000
Co2	0.0094 (4)	0.0105 (4)	0.0067 (4)	0.000	0.0038 (3)	0.000
As1	0.0077 (3)	0.0073 (3)	0.0047 (3)	0.000	0.0023 (2)	0.000
As2	0.0091 (2)	0.0071 (2)	0.0073 (2)	−0.00090 (15)	0.00172 (18)	0.00007 (17)
O1	0.0297 (19)	0.0157 (17)	0.015 (2)	0.0132 (15)	0.0073 (16)	−0.0024 (15)
O2	0.0091 (14)	0.0211 (18)	0.0157 (18)	0.0020 (13)	0.0018 (14)	−0.0030 (14)
O3	0.0093 (19)	0.015 (2)	0.008 (2)	0.000	0.0072 (18)	0.000
O4	0.0163 (14)	0.0152 (15)	0.0089 (15)	−0.0044 (13)	0.0061 (13)	−0.0053 (14)
O5	0.0128 (19)	0.011 (2)	0.008 (2)	0.000	−0.0008 (18)	0.000
O6	0.0114 (13)	0.0098 (14)	0.0158 (16)	0.0014 (12)	0.0064 (13)	0.0017 (13)
O7	0.014 (2)	0.040 (3)	0.004 (2)	0.000	0.005 (2)	0.000
Na1A	0.047 (6)	0.0192 (12)	0.021 (2)	0.012 (3)	0.011 (4)	−0.0034 (12)
Na1B	0.047 (6)	0.0192 (12)	0.021 (2)	0.012 (3)	0.011 (4)	−0.0034 (12)
Na2A	0.0179 (15)	0.0196 (17)	0.059 (3)	0.000	0.0126 (18)	0.000
Na2B	0.0179 (15)	0.0196 (17)	0.059 (3)	0.000	0.0126 (18)	0.000

### Geometric parameters (Å, º)

**Table:**

Co1—O1	2.108 (3)	Na1A—O6^viii^	2.621 (11)
Co1—O1^i^	2.108 (3)	Na1A—O2^viii^	2.646 (11)
Co1—O2	2.141 (3)	Na1A—O1^vii^	2.682 (13)
Co1—O2^i^	2.141 (3)	Na1A—O4^ix^	2.694 (10)
Co1—O6	2.177 (3)	Na1A—O7^viii^	2.782 (7)
Co1—O6^i^	2.177 (3)	Na1A—O6^vii^	2.936 (13)
Co2—O3^ii^	1.978 (4)	Na1B—O2	2.39 (2)
Co2—O7	2.003 (5)	Na1B—O2^viii^	2.47 (2)
Co2—O4^ii^	2.126 (3)	Na1B—O4^ix^	2.53 (2)
Co2—O4^iii^	2.126 (3)	Na1B—O7^viii^	2.75 (2)
Co2—O6^iv^	2.201 (3)	Na1B—O1^viii^	2.83 (2)
Co2—O6^v^	2.201 (3)	Na1B—O6^viii^	2.87 (2)
As1—O3	1.669 (4)	Na2A—O7	2.257 (5)
As1—O7	1.671 (5)	Na2A—O5	2.480 (4)
As1—O6^vi^	1.704 (3)	Na2A—O5^x^	2.480 (4)
As1—O6	1.704 (3)	Na2A—O4^xi^	2.501 (4)
As2—O1^vii^	1.670 (3)	Na2A—O4^x^	2.501 (4)
As2—O2	1.673 (3)	Na2B—O4^xi^	2.27 (6)
As2—O4	1.680 (3)	Na2B—O4^x^	2.27 (6)
As2—O5	1.790 (2)	Na2B—O4^ii^	2.30 (5)
Na1A—O2	2.475 (9)	Na2B—O4^iii^	2.30 (5)
Na1A—O1^viii^	2.540 (12)	Na2B—O7^xii^	2.51 (5)
			
O1—Co1—O1^i^	110.2 (2)	O3^ii^—Co2—O6^iv^	94.87 (13)
O1—Co1—O2	82.94 (14)	O7—Co2—O6^iv^	95.48 (14)
O1^i^—Co1—O2	90.01 (14)	O4^ii^—Co2—O6^iv^	93.63 (12)
O1—Co1—O2^i^	90.01 (14)	O4^iii^—Co2—O6^iv^	167.77 (12)
O1^i^—Co1—O2^i^	82.94 (14)	O3^ii^—Co2—O6^v^	94.87 (13)
O2—Co1—O2^i^	167.7 (2)	O7—Co2—O6^v^	95.48 (14)
O1—Co1—O6	81.33 (13)	O4^ii^—Co2—O6^v^	167.77 (12)
O1^i^—Co1—O6	168.19 (14)	O4^iii^—Co2—O6^v^	93.63 (12)
O2—Co1—O6	89.07 (13)	O6^iv^—Co2—O6^v^	74.22 (16)
O2^i^—Co1—O6	99.88 (13)	O3—As1—O7	101.9 (2)
O1—Co1—O6^i^	168.19 (14)	O3—As1—O6^vi^	115.27 (14)
O1^i^—Co1—O6^i^	81.33 (13)	O7—As1—O6^vi^	111.14 (15)
O2—Co1—O6^i^	99.88 (13)	O3—As1—O6	115.27 (14)
O2^i^—Co1—O6^i^	89.07 (13)	O7—As1—O6	111.14 (14)
O6—Co1—O6^i^	87.23 (18)	O6^vi^—As1—O6	102.4 (2)
O3^ii^—Co2—O7	167.01 (18)	O1^vii^—As2—O2	111.18 (19)
O3^ii^—Co2—O4^ii^	87.38 (12)	O1^vii^—As2—O4	114.04 (17)
O7—Co2—O4^ii^	84.15 (12)	O2—As2—O4	116.98 (16)
O3^ii^—Co2—O4^iii^	87.38 (12)	O1^vii^—As2—O5	103.39 (16)
O7—Co2—O4^iii^	84.15 (12)	O2—As2—O5	106.19 (13)
O4^ii^—Co2—O4^iii^	98.49 (17)	O4—As2—O5	103.43 (16)

Symmetry codes: (i) −*x*+1, *y*, −*z*+2; (ii) *x*, *y*, *z*−1; (iii) *x*, −*y*+1, *z*−1; (iv) −*x*+1, *y*, −*z*+1; (v) −*x*+1, −*y*+1, −*z*+1; (vi) *x*, −*y*+1, *z*; (vii) *x*−1/2, −*y*+1/2, *z*; (viii) −*x*+1/2, −*y*+1/2, −*z*+1; (ix) −*x*+1/2, −*y*+1/2, −*z*+2; (x) −*x*, −*y*+1, −*z*+1; (xi) −*x*, *y*, −*z*+1; (xii) −*x*, −*y*+1, −*z*.

### Bond-valence-sum and charge distribution analysis.

**Table:**

Cation	q(i).sof(i)	V(i)	Q(i)	CN(i)	ECoN(i)	d_moy_(i)	d_med_(i)
Co1	2.00	1.87	2.01	6	5.97	2.14	2.14
Co2	2.00	2.05	2.03	6	5.62	2.11	2.08
As1	5.00	4.99	5.13	4	3.99	1.69	1.69
As2	5.00	4.93	4.94	4	3.89	1.70	1.69
Na1A	0.72	0.65	0.72	8	7.33	2.67	2.63
Na1B	0.28	0.25	0.27	8	5.72	2.74	2.58
Na2A	0.94	1.02	0.92	7	5.52	2.58	2.45
Na2B	0.06	0.07	0.06	6	4.78	2.45	2.31

*q*(*i*) = formal oxidation number; *sof*(*i*) = site occupation factor; *d*_moy_(*i*) = arithmetic average distance; *d*_med_(*i*) = weighted average distance; sodium CNs for *d*(Na–O)_max_ = 3.10 Å; σ_cat_ = dispersion factor on cationic charges measuring the deviation of the computed charges (*Q*) with respect to the formal oxidation numbers; σ_cat_ = [Σ*_i_*(*q**_i_*-*Q**_i_*)^2^/*N*-1]^1/2^ = 0.055.
